# Thyrotoxic Periodic Paralysis (TPP): A Comprehensive Review with Regional Insights from the Middle East

**DOI:** 10.7759/cureus.98814

**Published:** 2025-12-09

**Authors:** Fahad S Alrashidi

**Affiliations:** 1 Internal Medicine/Nephrology, Ad Diriyah Hospital, Riyadh Third Health Cluster, Riyadh, SAU

**Keywords:** arrhythmia, emergency medicine, genetics, hyperthyroidism, hypokalemia, kcnj18/kir2.6, management, middle east, potassium rebound, thyrotoxic periodic paralysis

## Abstract

Background: Thyrotoxic periodic paralysis (TPP) is a rare but potentially life-threatening cause of acute flaccid paralysis, characterized by hypokalemia in the setting of thyrotoxicosis and often misdiagnosed in emergency departments.

Methods: We conducted a narrative literature review of TPP in adults using major databases up to 31 October 2025, with a focus on clinical presentation, precipitants, diagnostic pitfalls, and management, and we synthesized Middle Eastern case data alongside global experience.

Results: TPP typically affects younger males and may be the first manifestation of hyperthyroidism. High-carbohydrate meals, strenuous exercise, and β-agonists are common triggers. Misdiagnosis as Guillain-Barré syndrome or other neuromuscular conditions is frequent, particularly when thyrotoxicosis is subtle. Middle Eastern reports suggest similar clinical patterns but highlight delayed recognition and variable access to thyroid testing. Early correction of hypokalemia, non-selective beta (β)-blockade, and definitive control of thyrotoxicosis are essential to prevent recurrences.

Conclusions: An emergency department (ED)-focused approach that routinely considers TPP in acute flaccid paralysis, integrates rapid thyroid function testing, and follows structured potassium replacement and β-blocker protocols can reduce diagnostic delay and improve outcomes. Incorporating regional data may help tailor awareness campaigns and management pathways in Middle Eastern and other underrepresented settings.

## Introduction and background

Thyrotoxic periodic paralysis (TPP) is an uncommon, acute complication of thyrotoxicosis characterized by sudden flaccid muscle weakness due to a rapid intracellular shift of potassium, resulting in marked hypokalemia. Despite this relatively distinctive clinical triad, TPP frequently eludes timely recognition outside endemic settings [1]. Beyond the stereotypical young, otherwise healthy man with abrupt proximal weakness and profound hypokalemia, TPP can be misattributed to primary neurologic disease, renal potassium wasting, or toxicologic exposure. Global mobility, the rising prevalence of hyperthyroidism in diverse populations, and variable access to endocrine testing collectively argue for renewed attention to pragmatic diagnostic pathways that can be implemented in emergency departments (EDs) and acute care wards [2]. In the Middle East, where case reports document malignant arrhythmias and recurrent attacks before euthyroidism is achieved, a clinically oriented synthesis targeted to frontline clinicians is particularly warranted [3-7]. In this narrative review, we synthesize heterogeneous, predominantly case-based global and Middle Eastern literature to highlight practical bedside steps that may shorten time-to-diagnosis, standardize acute management, and reduce preventable morbidity, while acknowledging that the recommendations are derived from low-level evidence and should be interpreted as pragmatic practice suggestions rather than formal guideline directives.

## Review

Methods

We conducted a narrative literature review of TPP in adults, with a particular focus on clinical presentation, precipitants, diagnostic pitfalls, and management, and on synthesizing Middle Eastern case data within the broader global experience. This was not a registered systematic review or meta-analysis.

We searched three major electronic databases (PubMed, Scopus, and Web of Science) from database inception to 31 October 2025 (last search update). The core search strategy combined terms for the condition, thyroid status, and relevant clinical features, together with regional country terms. For example, the PubMed search used the Boolean structure, which was as follows: (“thyrotoxic periodic paralysis” OR “thyrotoxic hypokalemic periodic paralysis” OR “hypokalemic periodic paralysis”) AND (hyperthyroidism OR “Graves disease” OR thyrotoxicosis) AND (arrhythmia OR “beta-blocker” OR KCNJ18 OR Kir2.6) AND (Qatar OR “Saudi Arabia” OR Oman OR Bahrain OR “United Arab Emirates” OR Kuwait).

Similar combinations of these terms were adapted for Scopus and Web of Science using database-specific syntax [1-3]. Reference lists of key reviews and regional case reports were hand-searched to identify additional eligible articles. We included articles published in English, and for Middle Eastern cohorts, we also considered Arabic-language full texts when accessible. Non-English, non-Arabic vernacular reports, conference-only abstracts, and non-indexed local bulletins were not systematically searched, so some regional cases may not have been captured.

Eligibility Criteria and Study Selection

We included records that met all of the following criteria: (1) human subjects aged ≥18 years; (2) clear biochemical documentation of thyrotoxicosis at the time of the paralysis episode, or explicit linkage of the paralytic episodes to hyperthyroidism; and (3) provision of primary clinical data (case reports, case series, or observational cohorts) or clinically relevant syntheses (clinical practice guidelines, narrative reviews, or scoping reviews) addressing TPP. Reports that provided detailed electrolyte abnormalities and their management during acute episodes were prioritized.

We excluded pediatric-only series, animal or in vitro studies, editorials, and commentaries without primary data, studies in which thyroid status during the paralysis episode was not clearly documented, and duplicate publications from the same cohort unless they contributed non-overlapping additional information.

**Figure 1:**
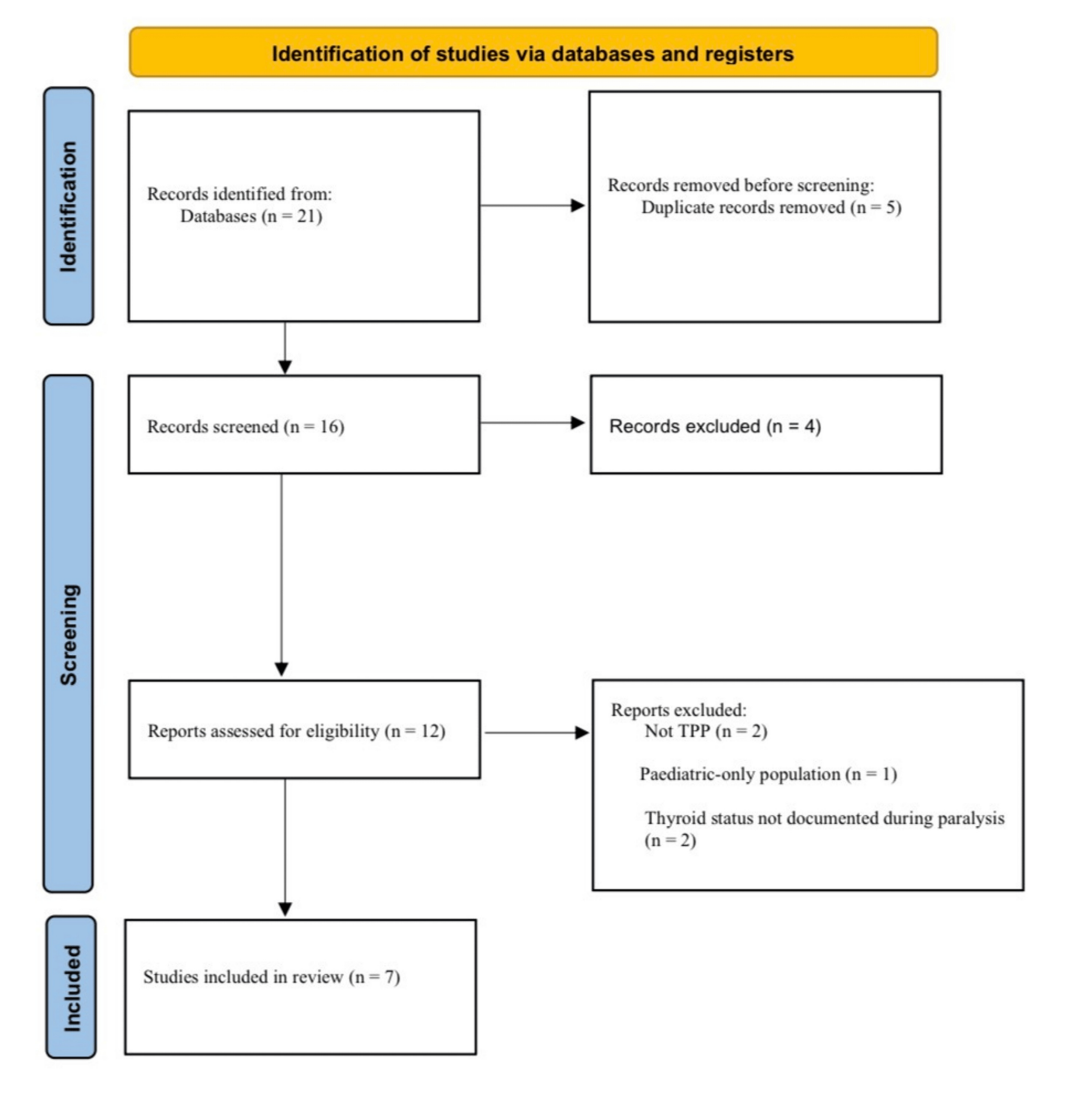
PRISMA 2020 flow diagram summarizing identification and selection of Middle Eastern TPP reports included in this narrative review. PRISMA: Preferred Reporting Items for Systematic Reviews and Meta-Analyses; TPP: thyrotoxic periodic paralysis

Titles and abstracts retrieved by the search were screened for relevance by the author. Potentially eligible articles underwent full-text review to confirm that they satisfied the inclusion and exclusion criteria described above. Because screening was conducted by a single reviewer, disagreements did not arise; ambiguous cases were resolved by repeat full-text review and cross-checking of biochemical data. Additional studies were identified by manually reviewing the reference lists of included articles and pertinent reviews. The overall selection process, including the number of records identified, screened, excluded (with main reasons), and included in the qualitative synthesis, is summarized in a Preferred Reporting Items for Systematic Reviews and Meta-Analyses (PRISMA) flow diagram (Figure 1).

Data Synthesis

Data from eligible reports were synthesized qualitatively using a thematic approach. Information was grouped into the following domains: epidemiology, pathophysiology, clinical features, diagnostic pitfalls, management strategies, and regional gaps. For observational cohorts, we qualitatively considered potential sources of bias (e.g., case definition, completeness of thyroid and electrolyte data, follow-up duration, and reporting of adverse events), but given that the evidence base is dominated by single case reports and small series, we did not apply a formal risk-of-bias tool.

Because randomized trials are lacking and available evidence largely comprises heterogeneous case reports, small series, and observational data, we did not attempt formal quantitative meta-analysis, pooled effect estimates, meta-regression, or calculation of confidence intervals. Where feasible, we report simple descriptive counts or proportions (for example, the distribution of cases by sex or country), but these are presented as approximate summaries rather than statistically pooled estimates. Recommendations in this review are therefore based on triangulation of clinical practice guidelines, mechanistic rationale, and repeatedly observed clinical patterns in the published literature and should be interpreted as pragmatic, consensus-style suggestions grounded in low-level evidence rather than as guideline-grade directives [4-7].

Results

Epidemiology

TPP shows striking geographic and ethnic variation, although reported frequencies are heavily influenced by heterogeneity in study design, case ascertainment, and diagnostic vigilance. In hyperthyroid cohorts from East Asia, TPP accounts for approximately 1.8%-1.9% of patients, whereas estimates from Western populations are much lower (~0.1%-0.2%); however, these figures are derived from single-center series with differing inclusion criteria and almost certainly underestimate the true burden in non-endemic settings [8-12]. Reports from high-volume Western centers describe frequent diagnostic delay, misattribution of hypokalemia to gastrointestinal or renal potassium losses, and potentially avoidable hospitalizations when TPP is not considered.

Within the Middle East, the published literature is limited to small case reports and series, totaling 27 adults across seven indexed publications from Qatar, Saudi Arabia, Oman, Bahrain, and the United Arab Emirates [13-18]. Despite the small numbers and likely publication bias, these reports describe a broadly consistent phenotype: predominantly young men presenting with acute flaccid weakness, profound hypokalemia, and few overt thyrotoxic stigmata at the bedside. Some Gulf series have noted that attacks appeared to occur more often during hotter months or after carbohydrate-rich meals and rest following exertion, raising the possibility that culturally influenced dietary patterns, activity cycles, and climate may modulate attack risk. Given the very small sample sizes and observational nature of these reports, any apparent “seasonal clustering” should be considered hypothesis-generating rather than definitive evidence of a climate-driven pattern. Recurrent episodes often precede definitive control of thyrotoxicosis, underscoring the importance of streamlined referral pathways for antithyroid drugs, radioiodine therapy, or surgery. From an implementation perspective, embedding thyroid function tests into emergency department hypokalemia order sets and using an ED-focused diagnostic algorithm may help shorten time to diagnosis and mitigate arrhythmia risk, but these approaches are proposed as pragmatic implementation suggestions based on low-level evidence rather than formal guideline recommendations and should be adapted to local resources and institutional protocols.

A summary of Middle Eastern TPP reports is provided in Table 1, which also illustrates the substantial clinical and methodological heterogeneity across these small, predominantly single-center series. These reports span different time periods, hospital settings, ethnic compositions, and depths of electrolyte and arrhythmia reporting, which limits direct comparability and precludes formal pooling but still allows recurring patterns to be appreciated.

**Table 1:** Published TPP cases/series in the Middle East N: number; TPP: thyrotoxic periodic paralysis; HPP: hypokalemic periodic paralysis

Country	Year	Journal and reference	Study type	N (TPP)	Key details
Qatar	2008	Netherlands Journal of Medicine [3]	Case series	18 (all male)	Mean age ≈32 years; summer clustering; high recurrence
Saudi Arabia	2009	Saudi Medical Journal [4]	Case report	1	K+ 1.5 mmol/L; transient asystole
Saudi Arabia	2016	Clinical Medicine Insights: Case Reports [5]	Case report	1	K+ 2.0 mmol/L; ventricular tachycardia and cardiac arrest; full recovery
Saudi Arabia	2017	BMJ Case Reports [6]	Case report	1	Initial presentation of Graves’ disease as TPP
Oman	2008	Oman Medical Journal [7]	Case report	1	K+ 2.6 mmol/L; no overt thyrotoxic signs
Bahrain	1998	Bahrain Medical Bulletin [8]	Case report	1	K+ 1.8 mmol/L; first report from Bahrain Defence Force Hospital
United Arab Emirates	2010	European Journal of Emergency Medicine [9]	Case series (hypokalemic periodic paralysis)	4 (of 17 HPP)	Male predominant; mostly Asian patients; seasonality

A comparison of regional patterns across global settings is shown in Table 2.

**Table 2:** Global comparison of TPP epidemiology TPP: thyrotoxic periodic paralysis

Region	Approximate TPP prevalence among hyperthyroid patients	Typical demographics	Notable triggers/Features	Representative sources
East Asia	~1.8–1.9%	Predominantly young men	High-carbohydrate meals; rest after strenuous exertion	Lin 2005 [10]; Kung 2006 [11]
Western countries	~0.1–0.2%	Predominantly young men; increasing recognition	Similar triggers; often initially misdiagnosed	Kung 2006 [11]
Middle East	Under-reported (likely lower than East Asia but higher than currently published)	Predominantly young men	Possible seasonal clustering; thyroid function tests often under-ordered in emergency departments	Regional case reports from Qatar, Saudi Arabia, Oman, Bahrain, and the United Arab Emirates [3–9]

Pathophysiology

TPP is best understood as a reversible redistribution of potassium into skeletal muscle rather than a large total-body deficit of potassium [19-23]. Excess thyroid hormone increases skeletal muscle Na⁺/K⁺-ATPase expression and activity and augments myocyte sensitivity to catecholamines and insulin. In this primed state, even modest adrenergic or insulin surges (for example, after strenuous exercise or a carbohydrate-rich meal) can trigger a brisk intracellular potassium shift, lower membrane excitability, and precipitate paralysis [24].

Genetic susceptibility appears to lower the threshold for this shift in a subset of individuals. Variants in inward-rectifier potassium channels such as Kir2.6 (encoded by KCNJ18) and related channelopathies have been reported in case-control and family studies, although findings are not uniform across populations, and such variants are neither necessary nor sufficient for TPP [1, 24-27]. Acid-base status (particularly alkalosis), sex hormones, and body composition further modulate cellular potassium uptake and are thought to contribute to the striking male predominance described in Asian and Middle Eastern cohorts [28-31].

Overall, these mechanisms provide a coherent explanatory framework for several characteristic clinical features of TPP, including (1) rapid improvement once adrenergic drive is blunted, for example, after non-selective beta (β)-blockade; and (2) the well-described risk of rebound hyperkalemia if aggressive potassium replacement is continued while the transcellular gradient is normalizing [25-27]. However, much of this mechanistic understanding is derived from small physiologic studies and case series, and individual patients may deviate from these patterns.

Clinical Features

Descriptions of TPP attacks across case series are broadly consistent but show some heterogeneity related to setting and case ascertainment. Attacks most often begin overnight or in the early morning, frequently after a high-carbohydrate evening meal or unaccustomed exertion, although clear triggers are not identified in all patients [18]. Weakness is typically symmetric and proximal, with flaccid tone, preserved sensation, and depressed or absent deep tendon reflexes. Respiratory and bulbar involvement are uncommon but may occur in severe episodes. In most reports, serum potassium ranges from approximately 1.5 to 3.0 mmol/L, and concomitant hypophosphatemia and hypomagnesemia are documented in a subset of cases. Thyroid function tests confirm underlying thyrotoxicosis, but the intensity of clinical thyrotoxic symptoms at presentation is highly variable.

Electrocardiographic abnormalities mirror the degree of hypokalemia and include sinus tachycardia, ST-segment depression, prominent U waves, and QT prolongation, warranting continuous monitoring for ventricular arrhythmias [5, 20,32-35]. Importantly, the absence of overt thyrotoxic signs should not preclude thyroid testing: several series highlight that patients may appear clinically euthyroid during attacks, leading to omission of thyroid studies and misclassification as primary renal or gastrointestinal potassium loss [35].

Key complications (including respiratory compromise and malignant arrhythmias) and practical monitoring priorities during acute episodes are summarized in Table 3.

**Table 3:** Reported complications and clinical implications

Complication	Mechanism/Association	Clinical implications
Ventricular tachycardia/Cardiac arrest	Severe hypokalemia leading to repolarization abnormalities; heightened adrenergic drive in thyrotoxicosis	Continuous cardiac monitoring; cautious intravenous potassium replacement; early use of non-selective beta-blockade when not contraindicated
Rebound hyperkalemia	Total-body potassium deficit is modest, and the transcellular shift reverses rapidly once triggers are controlled	Frequent electrolyte monitoring (e.g., every two to four hours initially); avoid over-replacement and discontinue potassium once weakness and ECG changes begin to resolve
Prolonged QT interval/Prominent U waves	Hypokalemia, often with concurrent hypomagnesemia	Correct magnesium and potassium; continuous telemetry; review and adjust QT-prolonging medications if possible
Respiratory muscle weakness (rare)	Severe generalized paralysis involving respiratory muscles in advanced attacks	Monitor vital capacity, respiratory rate, and gas exchange; early airway protection and ventilatory support if impending respiratory failure is suspected

Diagnostic Approach and Pitfalls

In the acute setting, typical initial priorities in patients presenting with flaccid weakness and hypokalemia include a 12-lead ECG with continuous cardiac monitoring and rapid serum chemistry assessment (potassium, magnesium, and phosphate), obtained in parallel with thyroid function tests (thyroid-stimulating hormone (TSH) with free thyroxine (T4) ± free triiodothyronine (T3)). These steps are widely used in emergency practice and allow simultaneous assessment of arrhythmia risk and identification of a potential thyrotoxic trigger. When diagnostic uncertainty persists, urine potassium indices can help distinguish a predominantly transcellular shift from renal potassium loss; a low urine potassium-to-creatinine ratio supports redistribution rather than true depletion, although treatment should not be delayed solely to obtain these measurements. Arterial or venous blood gas analysis may further clarify acid-base status, which can accentuate intracellular potassium uptake.

Several recurring pitfalls described in the case series warrant emphasis. One is the use of dextrose-containing resuscitation fluids, which stimulate insulin release and can aggravate hypokalemia. Another is the administration of large, unmonitored potassium chloride boluses, which increases the risk of rebound hyperkalemia once the transcellular shift reverses. Provocative testing is unnecessary; in routine practice, diagnosis rests on the combination of hypokalemia, biochemical thyrotoxicosis, a compatible clinical phenotype, and rapid improvement after non-selective β-blockade with cautious potassium repletion.

**Figure 2:**
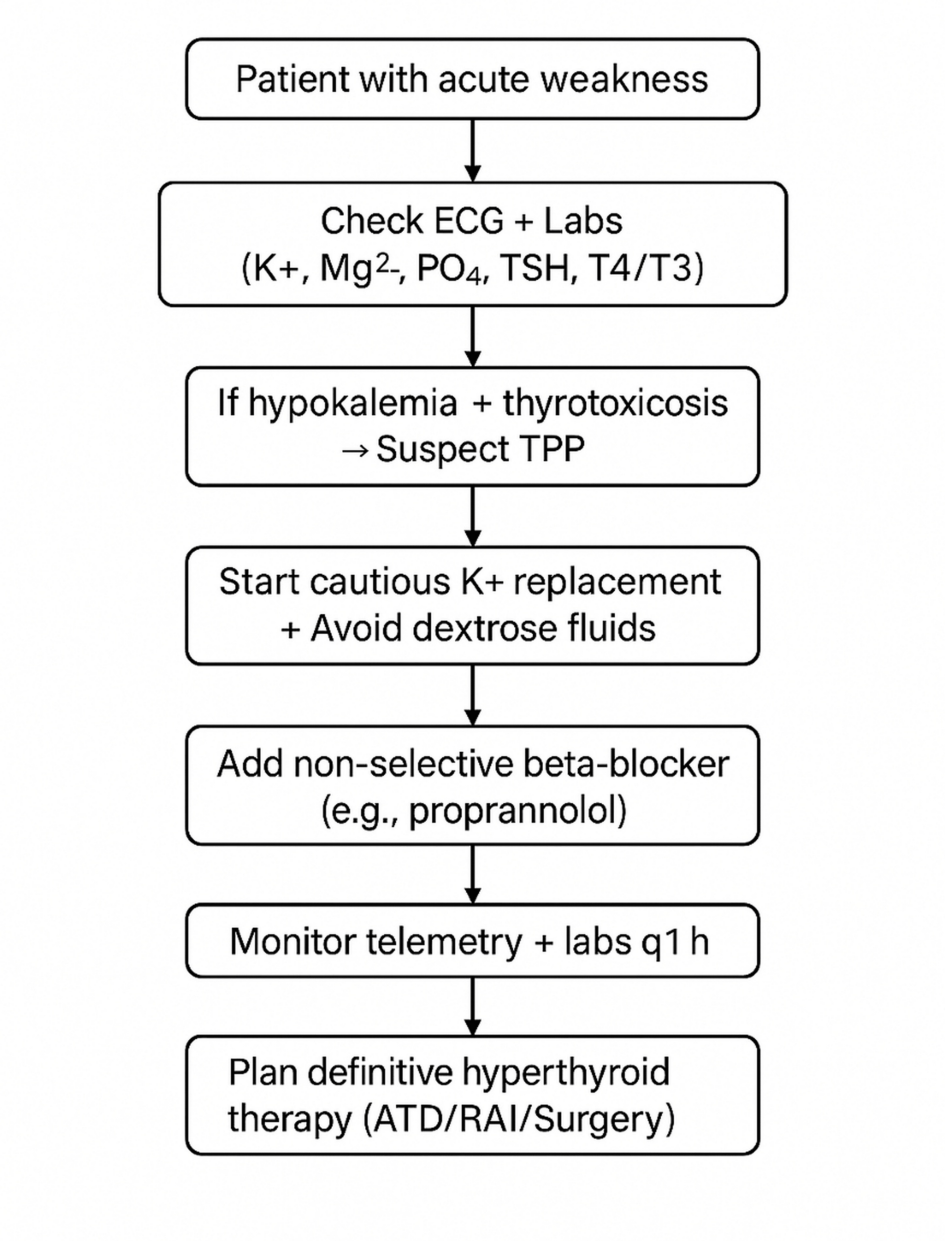
Emergency department algorithm for suspected TPP TPP: thyrotoxic periodic paralysis; K⁺: potassium; Mg²⁺: magnesium; PO₄: phosphate; TSH: thyroid-stimulating hormone; T3: triiodothyronine; T4: thyroxine; ATR: atrial tachyarrhythmia; RAI: radioactive iodine

A pragmatic, emergency department-focused diagnostic algorithm based on these principles is presented in Figure 2 [1, 24].

This algorithm is intended as an implementation aid and reflects commonly used practice patterns derived from case-based evidence and pathophysiologic reasoning, rather than a formal guideline.

Management

Acute management of TPP is directed at stabilizing the myocardium, correcting electrolyte abnormalities in a controlled manner, blunting adrenergic drive, and initiating definitive treatment of underlying thyrotoxicosis [1-3,9]. The specific strategies described below reflect commonly used practice patterns derived from case reports, small series, and hyperthyroidism guidelines, rather than randomized TPP-specific trials.

Definitive Control of Thyrotoxicosis

Antithyroid drug therapy (for example, methimazole) is often initiated in the ED or shortly thereafter to reduce the risk of further attacks. The choice among antithyroid drugs, radioiodine, and surgery should take into account disease severity, the presence of ophthalmopathy, comorbidities, patient preference, and access to reliable follow-up. Until a euthyroid state is achieved, structured counseling about potential triggers, such as large carbohydrate loads, vigorous unaccustomed exercise, alcohol, and over-the-counter sympathomimetics, may help curb recurrence risk. Many clinicians provide a brief written handout outlining foods and drinks to moderate and suggesting a gradual two to three week “ramp-up” in exercise intensity; while not formally studied, such pragmatic education is generally well-received and easy to implement.

*Non-selective β*-*Blockade*

Non-selective β-blockers attenuate catecholamine-driven Na⁺/K⁺-ATPase stimulation and reduce peripheral conversion of T4 to T3. Propranolol is the most frequently reported agent in the TPP series and in hyperthyroid practice more broadly. Commonly used oral regimens in the ED include 20-40 mg every six to eight hours, titrated to heart rate, blood pressure, and symptom relief; some reports describe intravenous dosing (for example, 1 mg over 10 minutes in a monitored setting), again with careful hemodynamic monitoring [4-6]. These doses are extrapolated from hyperthyroidism and thyroid storm management rather than from comparative trials in TPP and are presented as examples of typical practice rather than universally endorsed standards. Other nonselective or cardioselective β-blockers may also be appropriate, and agent choice and dosing should be guided by comorbidities, clinician preference, and local institutional protocols. In patients with asthma, decompensated heart failure, conduction disease, or other contraindications to β-blockade, risks and alternatives should be weighed carefully, and early consultation with cardiology and/or endocrinology is advisable. Where β-blockers are contraindicated, cautious potassium repletion with close telemetry may still reverse weakness, but durable prevention of recurrence continues to depend on rapid control of thyrotoxicosis.

Potassium Replacement and Monitoring

For hemodynamically stable patients who can take oral medications and have no malignant arrhythmias, oral potassium chloride in divided doses is generally preferred. Many clinicians start with a total of 20-40 mmol (for example, 10-20 mmol every one to two hours), with reassessment of muscle strength, serum potassium, and telemetry at each interval. Because the predominant disturbance in TPP is a transcellular shift rather than a large whole-body potassium deficit, cautious titration is usually sufficient to restore neuromuscular function without exceeding renal excretory capacity.

Intravenous potassium is typically reserved for patients with severe paralysis, bulbar or respiratory compromise, marked electrocardiographic abnormalities, or inability to tolerate oral intake. Conservative initial rates around 10 mmol/h under continuous cardiac monitoring are commonly used; escalation toward 20 mmol/h is generally restricted to central venous access in high-acuity settings and should follow local institutional protocols. Concomitant hypomagnesemia and hypophosphatemia are frequently reported and merit correction, as each can perpetuate membrane instability and delay recovery [4,5,7]. Glucose-containing fluids are usually avoided in the acute phase to prevent insulin-mediated worsening of hypokalemia. These dose ranges and rate limits should be interpreted as examples of commonly used practice patterns rather than rigid protocol rules and must be adapted to local guidelines and monitoring capabilities.

Preventing Rebound Hyperkalemia and Planning Discharge

Because total-body potassium deficit in TPP is typically modest and the transcellular shift reverses rapidly once triggers are removed and β-blockade is established, over-replacement can result in rebound hyperkalemia. Once motor strength improves and the serum potassium approaches approximately 3.0-3.5 mmol/L, potassium supplementation is often tapered or temporarily paused, with repeat laboratory assessment within 30-60 minutes to confirm stabilization.

Patients considered for ED discharge should have clear written and verbal instructions on trigger avoidance, warning symptoms (such as recurrent weakness, palpitations, or dyspnea), and indications for urgent reassessment, as well as a concrete plan for rapid endocrine follow-up [1,4,8]. Alignment between these pragmatic measures and existing hyperthyroidism guidelines, together with suggested ED- and ward-based practices, is summarized in Table 4.

**Table 4:** Guideline alignment and practical recommendations in TPP TPP: thyrotoxic periodic paralysis; T3: triiodothyronine; T4: thyroxine; β: beta

Aspect	Guideline alignment/Evidence-based	Practical recommendations for ED and acute care
Thyroid testing in hypokalemia	International hyperthyroidism and endocrine emergency guidance emphasizes considering thyrotoxicosis in unexplained hypokalemia with muscle weakness.	Include TSH and free T4 (± free T3) in order sets for ED patients presenting with acute flaccid weakness and unexplained hypokalemia.
Potassium replacement strategy	Reviews of TPP and electrolyte guidelines highlight that total-body potassium deficit is usually modest and that aggressive replacement can cause rebound hyperkalemia.	Use cautious, incremental potassium replacement (e.g., 10–20 mmol doses with reassessment) rather than large boluses; reserve IV replacement and higher infusion rates for severe cases with continuous telemetry.
Avoidance of dextrose-containing fluids	TPP reviews consistently warn that glucose-containing fluids may worsen hypokalemia by stimulating insulin-mediated intracellular potassium shift.	Avoid dextrose-containing infusions in the acute phase; use isotonic, glucose-free solutions unless there is a compelling indication for dextrose.
Non-selective β-blockade	Hyperthyroidism and TPP guidance support non-selective β-blockers (e.g., propranolol) to blunt adrenergic drive and reduce T4-to-T3 conversion.	Initiate a non-selective β-blocker early (when not contraindicated), titrating to heart rate and clinical response; seek cardiology input in patients with heart failure or other relative contraindications.
Monitoring for arrhythmias	Electrolyte and endocrine emergency guidance recommend continuous monitoring in severe hypokalemia and in patients at risk of ventricular arrhythmias.	Place patients on continuous ECG monitoring until potassium has normalized, ECG changes have resolved, and muscle strength has clearly improved.
Correction of coexisting electrolyte abnormalities	Electrolyte management guidelines note that hypomagnesemia and hypophosphatemia can perpetuate arrhythmia risk and muscle weakness.	Check and correct magnesium and phosphate alongside potassium to optimize membrane stability and neuromuscular recovery.
Definitive control of thyrotoxicosis	Hyperthyroidism guidelines emphasize that restoration of euthyroidism is essential to prevent recurrence of TPP and long-term complications.	Arrange early endocrine follow-up to initiate or optimize antithyroid therapy, and to plan definitive treatment (continued antithyroid drugs, radioiodine, or surgery) based on patient profile and local practice.
Patient education and trigger avoidance	Patient-centered guidance and case series stress the importance of counseling on modifiable triggers between attacks.	Provide simple, written advice on moderating carbohydrate binges, pacing vigorous exercise, avoiding unnecessary sympathomimetics and excess alcohol, and seeking urgent care if recurrent weakness, palpitations, or dyspnea occur.

Special Populations and Practical Nuances

Evidence specific to TPP in special populations is scarce, and most recommendations are extrapolated from general hyperthyroidism and electrolyte-management guidelines, together with isolated case reports. The points below are therefore intended as pragmatic considerations rather than population-specific protocols.

Pregnancy and lactation: In pregnancy and lactation, the choice of antithyroid therapy must balance maternal-fetal safety with timely control of thyrotoxicosis. Management of the underlying thyroid disorder generally follows standard obstetric endocrinology guidance, with TPP viewed as an acute complication of that underlying disease. When β-blockers are required for acute control, the lowest effective dose for the shortest possible duration is usually preferred, with obstetric input where feasible. In the peripartum period, careful attention to the composition and rate of intravenous fluids and avoidance of unnecessary dextrose loads may help reduce the likelihood of precipitating attacks, although this has not been formally studied in TPP. Breastfeeding mothers benefit from clear counseling on medication safety, trigger avoidance, and the importance of maintaining scheduled endocrine follow-up.

Chronic kidney disease (CKD): In patients with CKD, profound hypokalemia from a transcellular shift can still occur despite impaired renal excretion. This combination can make potassium replacement particularly hazardous: even modest supplementation may lead to overshoot once the intracellular shift reverses. Telemetry-guided, incremental potassium dosing with frequent reassessment of serum electrolytes and acid-base status is therefore essential, and local institutional protocols for potassium administration in CKD should be followed closely. In advanced CKD or dialysis patients, early involvement of nephrology can assist with tailoring replacement strategies and considering dialysate potassium adjustments where appropriate.

Diabetes and metabolic syndrome: In individuals with diabetes or metabolic syndrome, postprandial insulin excursions may be pronounced and could lower the threshold for TPP attacks in the setting of untreated thyrotoxicosis. Observational reports suggest that many such patients present after large carbohydrate loads, but systematic data are lacking [15]. Individualized counseling on carbohydrate intake, review of glucose-lowering regimens (including insulin and insulin secretagogues), and early endocrinology follow-up are particularly valuable in this group. Coordinated management of both thyroid status and glycemic control is likely to be important for long-term prevention, although this has not been specifically evaluated in TPP-focused trials.

Differential Diagnosis

Several conditions can mimic the presentation of TPP and should be considered in the differential diagnosis of acute flaccid weakness with hypokalemia. Familial hypokalemic periodic paralysis typically presents earlier in life, often during childhood or adolescence, may have a positive family history, and is characterized by normal thyroid function tests. Renal potassium losses, for example, due to diuretics, mineralocorticoid excess, or tubular disorders, are suggested by inappropriately high urine potassium excretion in the appropriate clinical context, although overlap with TPP can occur if more than one mechanism is present. Gastrointestinal losses (vomiting, diarrhea, laxative abuse) are usually evident from the history but may coexist with thyrotoxicosis.

Iatrogenic and toxicologic intracellular shifts, such as those induced by insulin, β-agonists, or theophylline, can often be identified through careful medication review and exposure history [21]. Neuromuscular disorders (for example, Guillain-Barré syndrome, myasthenia gravis, or acute myelopathies) may present with flaccid weakness but are distinguished by the absence of significant hypokalemia and by characteristic neurologic findings. In practice, the combination of acute hypokalemic paralysis, biochemical thyrotoxicosis, compatible triggers, and rapid improvement after non-selective β-blockade with cautious potassium repletion strongly favors TPP, whereas discrepancies in this pattern should prompt broader evaluation. Distinguishing clinical and biochemical features of key differential diagnoses is summarized in Table 5.

**Table 5:** Differential diagnosis and distinguishing features TPP: thyrotoxic periodic paralysis; TSH: thyroid-stimulating hormone; T3: triiodothyronine; T4: thyroxine; K⁺: potassium

Condition	Typical clinical context	Thyroid status	Urine K⁺/Acid–base profile	Key distinguishing features
Thyrotoxic periodic paralysis (TPP)	Young adult, often male; attacks of painless flaccid weakness, frequently after high-carbohydrate meals or rest following exertion; subtle or previously unrecognized hyperthyroid symptoms may be present.	Overt thyrotoxicosis: suppressed TSH with elevated free T4 ± free T3.	Serum K⁺ typically 1.5–3.0 mmol/L; urine K⁺/creatinine ratio relatively low for the degree of hypokalemia; acid–base status often normal or mildly alkalotic.	No family history of periodic paralysis; features of hyperthyroidism on review; rapid improvement with non-selective β-blockade and cautious K⁺ repletion; recurrence prevented by restoration of euthyroidism.
Familial hypokalemic periodic paralysis	Onset in childhood or adolescence; recurrent episodes of flaccid weakness often triggered by rest after exercise or carbohydrate load; frequently positive family history.	Thyroid function tests are normal between and during attacks.	Serum K⁺ low during attacks; urine K⁺/creatinine ratio generally low; acid–base status usually normal or mildly alkalotic.	Autosomal dominant channelopathies (for example, CACNA1S, SCN4A) in many families; no biochemical thyrotoxicosis; similar triggers but earlier age of onset and strong family history.
Renal potassium-wasting disorders	History of diuretic use, mineralocorticoid excess, or tubular disorders (for example, Bartter or Gitelman syndromes); may be accompanied by hypertension or long-standing polyuria.	Thyroid function usually normal unless there is a separate thyroid disorder.	Inappropriately high urinary K⁺ excretion for the degree of hypokalemia; often metabolic alkalosis and elevated urinary chloride.	Persistent hypokalemia rather than discrete attacks; supporting clues from blood pressure, renin–aldosterone profile, diuretic history, and chronic metabolic alkalosis.
Gastrointestinal potassium loss	History of vomiting, nasogastric suction, diarrhea, or laxative abuse; clinical signs of volume depletion may be present.	Thyroid function tests are typically normal.	Urine K⁺ usually low in the setting of hypokalemia; metabolic alkalosis common with vomiting/nasogastric suction, whereas metabolic acidosis may be seen with severe diarrhea.	Clear history of ongoing GI fluid loss; symptoms of volume depletion; no clinical or biochemical evidence of thyrotoxicosis.
Drug- or toxin-induced intracellular shift	Temporal association with insulin boluses, β-agonists, theophylline, barium exposure, or other agents that promote cellular K⁺ uptake.	Thyroid status is usually normal; hyperthyroidism, if present, is not required for the episode.	Serum K⁺ low with relatively low urinary K⁺ excretion; acid–base status variable and depends on the underlying condition and coexisting therapies.	Close temporal relationship between drug/toxin exposure and onset of weakness; resolution as the pharmacologic effect wanes; absence of sustained thyrotoxicosis or family history of periodic paralysis.

Regional Insights, Implementation, and Research Gaps

Implementation-focused approaches may help translate existing TPP knowledge into routine practice in the Middle East, although formal evaluations are lacking. At the hospital level, thyroid testing could be embedded into electronic hypokalemia order sets, and emergency departments might adopt standardized, locally adapted algorithms that outline indications for telemetry, cautious potassium replacement, and early consideration of non-selective β-blockade. Clear pathways for expedited endocrinology follow-up are essential to facilitate timely initiation and optimization of definitive hyperthyroid therapy, irrespective of the specific modality chosen.

At the health system level, a regional registry or coordinated multicenter dataset could help clarify incidence, precipitant patterns, and outcomes, while enabling simple quality metrics such as time to diagnosis, length of stay, and readmission rates. Prospective audits before and after implementation of standardized ED and ward protocols might document changes in the frequency of recurrent attacks or arrhythmia events and could inform iterative refinement of care pathways. Given potential cultural and seasonal influences on diet and activity, including Ramadan-related shifts in meal timing and composition, context-aware patient education materials tailored to local practice may further mitigate recurrence risk, although these strategies have not yet been rigorously evaluated [1,26].

In practical terms, potential implementation priorities for the region may include embedding thyroid testing in ED hypokalemia order sets, developing regionally adapted ED algorithms for suspected TPP, providing targeted clinician education on diagnostic pitfalls and common triggers, and establishing reliable, expedited referral pathways to endocrine services. These examples are proposed as pragmatic implementation strategies based on low-level evidence and clinical experience, rather than as formal guideline mandates, and should be adapted to local resources and institutional protocols. Important research gaps remain, including the need for multicenter registries, prospective audits of time to diagnosis and recurrence, standardized reporting frameworks, genetic studies in Middle Eastern populations, and formal assessment of Ramadan-related and other culturally patterned triggers. Together, such efforts may help move the field from anecdote-based management toward more systematically evaluated care models.

Limitations of the Evidence

The current evidence base for TPP is dominated by case reports and small observational series, with a paucity of high-quality prospective data. Publication bias likely inflates the apparent frequency of dramatic presentations such as malignant arrhythmias and cardiopulmonary arrest, whereas milder, self-limited, or misdiagnosed episodes may go unreported, particularly in resource-limited or non-English-speaking settings. Heterogeneity in study design, inclusion criteria, and reporting practices further complicates attempts to derive precise incidence estimates or robust risk stratification for arrhythmic and respiratory complications. As a result, the true impact of specific behavioral triggers, comorbidities, and genetic variants remains uncertain.

In addition, our structured database search was restricted to English-language publications, with only opportunistic inclusion of indexed Arabic-language reports. Non-English, non-Arabic vernacular publications, conference-only abstracts, and non-indexed local bulletins from Middle Eastern centers were not systematically searched and are therefore likely underrepresented. This language and indexing bias may underestimate the true number of regional TPP cases and limit the generalizability of our regional observations.

Randomized trials are lacking, and very few studies have systematically evaluated standardized ED pathways or implementation strategies in terms of clinically meaningful outcomes. Most available data are drawn from single-center experiences without control groups, further limiting external validity. Consequently, the recommendations in this review should be understood as pragmatic, consensus-based practice suggestions that rely largely on triangulation of pathophysiologic reasoning, hyperthyroidism guideline statements, and recurring case-based observations, rather than on high-level prospective or randomized evidence. Clinicians should therefore adapt these suggestions to local resources, institutional protocols, and individual patient factors, and future research should aim to test such approaches prospectively wherever feasible.

## Conclusions

In summary, TPP is an uncommon but potentially highly reversible cause of acute flaccid paralysis when it is promptly recognized and managed. A practical, regionally informed approach that combines standardized ED-oriented diagnostic pathways, cautious potassium repletion, early consideration of non-selective β-blockade, and timely definitive treatment of thyrotoxicosis may help reduce preventable morbidity and arrhythmic complications, although these strategies have not been formally tested in randomized trials. Embedding thyroid testing within hypokalemia workflows and developing multicenter collaborations and registries across the Middle East are realistic initial steps toward closing the recognition gap and generating more robust prospective data to guide future practice.
